# Anévrysme de l'aorte ascendante associé à une insuffisance aortique massive: complication rare et grave de la maladie de Behçet

**DOI:** 10.11604/pamj.2015.21.85.6076

**Published:** 2015-06-03

**Authors:** Abdelkhalek Chetoui, Hicham El Malki, Mohamed Bahous, Jaafar Rhissassi, Rochde Sayah, Mohamed Laaroussi

**Affiliations:** 1 Service de Cardiologie A, CHU Ibn Sina, Rabat, Maroc; 2 Service de Chirurgie Cardiovasculaire A, CHU Ibn Sina, Rabat, Maroc

**Keywords:** Maladie de Behçet, anévrysme de l´aorte, insuffisance aortique, Behçet disease, aortic aneurysm, aortic insufficiency

## Abstract

L'atteinte artérielle au cours de la maladie de Behçet survient chez 2 à 12% des patients et se traduit par des lésions oblitérantes et/ou anévrysmales prédominant sur les gros troncs. Les complications cardiaques sont plus rares (1 à 6%) touchant les trois tuniques. En revanche, les anévrysmes de l'aorte ascendante associés à une insuffisance aortique restent une complication très rare de la maladie de Behçet. Nous rapportons l'observation d'un jeune patient de 35ans suivie pour une maladie de Behçet compliquée d'un anévrysme de l'aorte ascendante associé à une régurgitation aortique massive. Le diagnostic a été posé sur les données cliniques radiologiques de l’échocardiographie et de la tomodensitométrie puis confirmé à l'examen histologique de la pièce. Le traitement était chirurgical et a consisté en un remplacement total de la racine de l'aorte à cœur ouvert selon la technique de Bentall afin d’éviter le risque de rupture ou de dissection. L’évolution à 18 mois de l'intervention était favorable. Le traitement médical associant la corticothérapie et les immunosuppresseurs est la règle en postopératoire pour éviter les récidives.

## Introduction

La maladie de Behçet est une vascularite dont l’étiologie demeure encore inconnue malgré les hypothèses étiopathogéniques figurant dans la littérature. L'atteinte vasculaire est fréquente et peut se présenter sous des formes très variées, 37% d'atteintes veineuses,12% d'atteintes artérielles et 6% de manifestations cardiaques [1]. L'anévrysme de l'aorte ascendante associé à une insuffisance de la valve aortique est une complication très rare mais grave de la maladie de Behçet [2]. Nous rapportons le cas d'un anévrysme de l'aorte ascendante associé à une régurgitation aortique massive compliquant une maladie de Behçet chez un jeune patient opéré avec succès à cœur ouvert selon la technique de Bentall.

## Patient et observation

**Figure 1:**
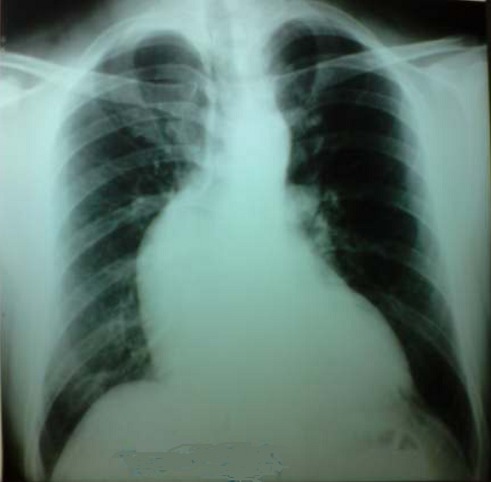
Radiographie du thorax: élargissement médiastinal

**Figure 2:**
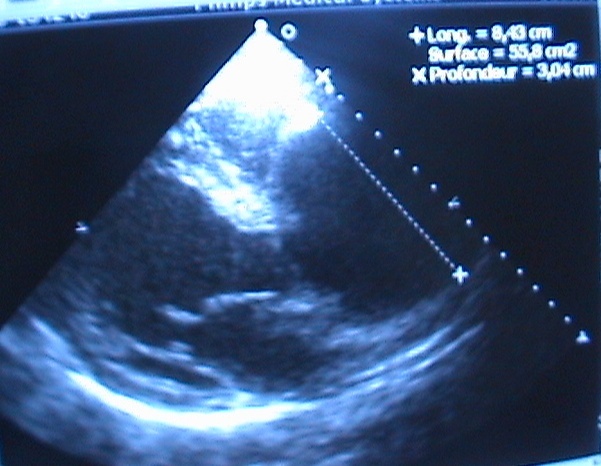
Coupe parasternale, grand axe, montrant la dilatation de l'aorte ascendante

**Figure 3:**
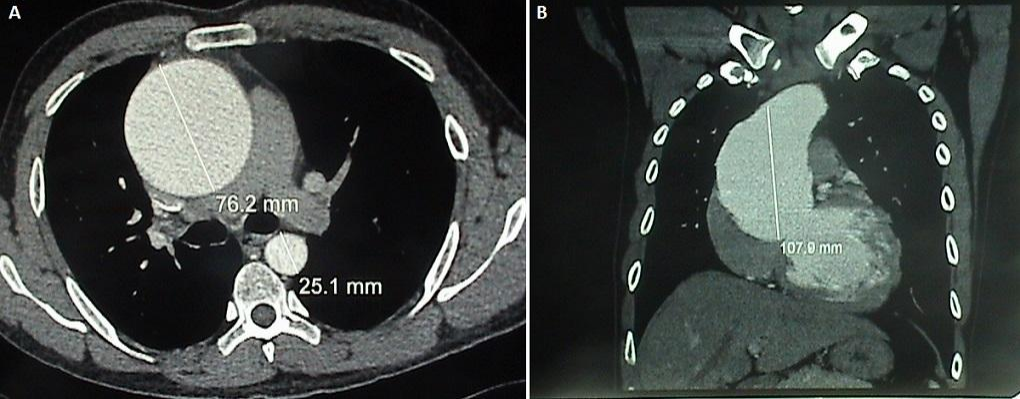
(A): angioscanner thoracique(coupe transversale): dilatation anévrismale de l'aorte ascendante; (B): angioscanner thoracique (coupe sagitalle): Dilatation anévrismale de l'aorte ascendante

**Figure 4:**
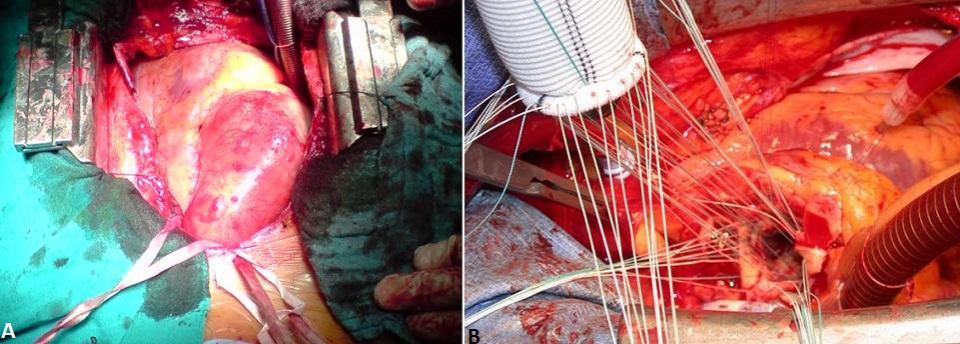
(A): dilatation anévrismale de l'aorte ascendante: vue peropératoire; (B): vue opératoire montrant le remplacement de la racine de l'aorte par un tube valvé

Il s'agit d'un jeune homme marocain de 35ans suivi pour maladie de Bechet depuis l’âge de 22 ans évoluant par poussées traitées par colchicine et qui consulte pour une dyspnée d'effort stade II de la NYHA (New York Heart Association). L'examen clinique a trouvé un patient eupnéique au repos, satension artérielle étaità 140/60 mm Hg, ses pouls périphériques étaient amples et bondissants avec à l'auscultation cardiaque un souffle diastolique d'insuffisance aortique coté 2/6^ème^ au foyer aortique. L'examen cutanéo-muqueux a montré des cicatrices d'aphtose scrotale et de pseudo-folliculites du tronc. L’électrocardiographe a objectivé une hypertrophie ventriculaire gauche de type diastolique. La radiographie du thorax a montré un élargissement médiastinal avec un indice cardiothoracique à 0.4 (Figure 1). L’échocardiographie transthoracique a visualisé un ventricule gauche dilaté à parois hypertrophiées et à fonction systolique conservée avec une fraction d’éjection estimée à 55%, une insuffisance aortique massive avec dilatation anévrysmale du Sinus Valsalvaet de l'aorte ascendante (Figure 2). L'angioscanner thoracique a objectivé une dilatation anévrysmale de l'aorte ascendante mesurant 76 mm de diamètre et du Sinus de Valsalva mesurant 42 mm (Figure 3). L'angioscanner cérébral et abdominal, ainsi que l’échodoppler des membres inférieurs n'ont pas révélés d'autres localisations anévrysmales. Le bilan inflammatoire a été normal: le patient a été en phase de rémission. L'indication opératoire est retenue devant l'importance de l'anévrysme et de la fuite aortique afin de prévenir le risque de rupture ou de dissection. Le geste chirurgical a consisté en un remplacement total de la valve aortique, de la racine de l'aorte ascendante avec réimplantation des artères coronairesselon la technique de Bentall modifiée sous circulation extra-corporelle (CEC) (Figure 4). L’étude anatomopathologique de la pièce opératoire a confirmé le diagnostic étiologique (la média, l'adventice et le pourtour des vasa vasorum ont été le siège d'un infiltrat riche en polynucléaires neutrophiles, lymphocytes et plasmocytes avec quelques éosinophiles et parfois des cellules géantes, évoquant la maladie de Behçet). Les suites opératoires ont été simples et l’évolution à court et à moyen terme a été favorable.

## Discussion

La maladie de Behçet est une maladie systémique dont le tropisme vasculaire se manifeste essentiellement par des phlébites à tel point qu'elles peuvent être considérées comme un signe cardinal de la maladie [1, 3]. Cette maladie affecte préférentiellement les sujets jeunes avec nette prédominance masculine. L'atteinte artérielle se traduit par des lésions oblitérantes et/ou anévrysmales prédominant sur les gros troncs mais pouvant également touché les artères périphériques [4–6]. L'atteinte cardiovasculaire apparait dans 2 à 28% des cas. Les lésions artérielles anévrismales sont présentes dans 65% des cas [7]. L'insuffisance aortique est découverte dans 5 à 19% des cas et constitue avec la dilatation anévrysmale de l'aorte ascendante les principales causes de décès au cours de l'angio-Behçet par rupture ou dissection. L'anévrysme est dû à une destruction de la paroi artérielle avec panvascularite et présence à la phase aiguë d'un infiltrat inflammatoire lymphocytaire envahissant particulièrement la média et l'adventice associé une oblitération des vasa vasorum, responsable d'une faiblesse pariétale pouvant aboutir à la formation d'un anévrysme ou d'une perforation, un véritable aphte vasculaire. Le diagnostic repose essentiellement sur l’échocardiographie et l'Angioscanner. La chirurgie demeure le seul traitement curatif [1, 7]. Le remplacement de la racine aortique requiert certaines précautions chirurgicales afin de prévenir la désinsertion prothétique secondaire et l'apparition de faux anévrismes qui sont assez fréquents [8, 9]. Plusieurs approches chirurgicales ont été proposées à cet effet, la mise en place d'un tube valvé (intervention de Bentall) et l'homogreffe aortique sont les plus utilisés. Outre ces différentes techniques d'autres précautions sont à entreprendre pour prévenir les complications postopératoires, une phase de rémission clinique et biologique est recommandée en préopératoire car elle conditionne les suites ultérieures postopératoires [10]. De même, les malades opérés dans un contexte d'urgence en phase active de la maladie doivent bénéficier d'un traitement anti inflammatoire précoce en postopératoire en dépit de son effet immunosuppresseur pouvant favoriser les infections et le retard de cicatrisation de la plaie opératoire [10–12].

## Conclusion

Les patients souffrant de la maladie de Behçet doivent bénéficier d'un dépistage précoce des atteintes vasculaires. L'atteinte anévrismale de l'aorte ascendante est rare et de mauvais pronostic. Le traitement chirurgical reste le traitement de choix pour éviter la rupture anévrismale. La surveillance clinique et radiologique à long terme est justifiée par le risque de récidive.
